# Fetal sex and the development of future type 2 diabetes: a 5-year cohort study

**DOI:** 10.1007/s00404-025-08125-0

**Published:** 2025-09-01

**Authors:** Liron Livny, Mordechai Hallak, Amir Naeh, Yoel Toledano, Rinat Gabbay-Benziv, Esther Maor-Sagie

**Affiliations:** 1 Technion-Israel Institute of Technology, Haifa, Israel; 2 Department of Obstetrics and Gynecology, Hillel Yaffe Medical Center, 38100 Hadera, Israel; 3 Meuhedet HMO, Tel Aviv, Israel

**Keywords:** Type 2 diabetes mellitus, Fetal gender, Gestational diabetes, Pregnancy, Fetal sex, Diabetes progression

## Abstract

**Purpose:**

Gestational Diabetes Mellitus (GDM) increases the risk of developing Type 2 Diabetes Mellitus (T2DM) postpartum, with emerging evidence suggesting that fetal sex may influence pregnancy outcomes. Some studies suggest that individuals carrying male fetuses experience diminished insulin sensitivity and higher glucose levels during pregnancy. However, it remains unclear whether fetal sex affects the long-term risk of T2DM after pregnancy. This study aimed to investigate the impact of fetal sex on the risk of T2DM up to 5 years postpartum in individuals with GDM.

**Methods:**

This retrospective analysis included pregnant individuals diagnosed with GDM, with follow-up data from Meuhedet HMO’s computerized pregnancy registry and the Israeli National Diabetes Registry. Inclusion criteria involved singleton pregnancies with GDM diagnosed via abnormal oral glucose tolerance tests (oGTT) or glucose challenge tests (GCT). Exclusion criteria were multifetal pregnancies or prior diabetes diagnosis. Maternal characteristics, obstetrics data, and T2DM incidence were compared by fetal sex using univariate and survival analyses, adjusted for confounders.

**Results:**

A total of 1637 individuals with GDM were included, with 808 carrying male fetuses and 829 carrying female fetuses. No significant differences were found in body mass index (BMI), glucose levels, or T2DM incidence between the two groups (6.2% for male vs. 7.1% for female fetuses, p = 0.48). Multivariate analysis identified maternal age and BMI as significant factors influencing the likelihood of developing T2DM.

**Conclusion:**

The risk of developing T2DM after GDM does not vary based on fetal sex.

## What does this study add to the clinical work

**Table:** 

This large-scale, population-based study leverages extensive data from two nationwide registries to assess the relationship between fetal sex and the risk of developing type 2 diabetes mellitus (T2DM) following gestational diabetes mellitus (GDM). Contrary to some previous findings, our results indicate that fetal sex does not significantly impact the progression to T2DM up to five years postpartum.	

## Introduction

Gestational diabetes mellitus (GDM) represents a significant medical concern during pregnancy, affecting approximately 7% of births in the United States in 2020 [1] and demonstrating a wide global prevalence ranging from 2 to 38%, influenced by diverse population demographics and diagnostic criteria [2]. Beyond its immediate implications for maternal and fetal health, GDM has been linked to an elevated risk of type 2 diabetes mellitus (T2DM) in the mother, with studies suggesting that as many as 70% of women diagnosed with GDM may develop diabetes within 22–28 years postpartum [3, 4].

The relationship between fetal sex and the development of diabetes during and after pregnancy has emerged as an area of growing interest. Previous research has indicated potential variations in pregnancy outcomes based on the sex of the fetus [5–7]. Specifically, some studies have shown an association between male fetus and poor β-cell function, higher postprandial glycemia, and an increased risk of GDM in the mother [7–9]. Others have suggested an elevated risk of T2DM following pregnancies with female fetuses [10].

Given the complexity of these associations, our study aims to investigate the risk of progression to T2DM following GDM, stratified by fetal sex, up to 5 years postpartum. This research seeks to provide deeper insights into how fetal sex may influence the trajectory of diabetes risk in women with a history of GDM, thereby contributing to our understanding of personalized preventive strategies and healthcare interventions for at-risk populations.

## Materials and methods

A retrospective study aimed to evaluate the risk of progression to T2DM development following singleton pregnancy with GDM according to fetal sex. The Meuhedet Institutional Review Board Committee (REB-10-18-08-21) approved the study. Due to the retrospective nature of the study, informed consent was waived.

### Study population

In this study, we utilized an extensive dataset spanning over 5 years of pregnancy registry and laboratory data collected by the Meuhedet Health Maintenance Organization (HMO). Meuhedet is one of the four state-mandated health funds that all Israeli residents must belong to under the country’s universal healthcare system. This dataset was crossed with the Israeli National Diabetes Registry (INDR) to evaluate the risk of developing T2DM.

The dataset included all pregnant individuals with documented pregnancies (per the pregnancy registry) whose last menstrual period (LMP) fell between January 1, 2017, and December 31, 2020. Maternal data encompassed maternal age, body mass index (BMI), hypertension diagnosis, hypertriglyceridemia, and hypercholesterolemia. Delivery data included gestational age at delivery and neonatal sex. All clinical data were retrieved from the parturient’s electronic medical records during pregnancy, with BMI calculated based on maternal weight and height measured at the first medical encounter during pregnancy. Laboratory data comprised first-trimester fasting plasma glucose levels, 50 g GCT results, and 100 g OGTT) values. T2DM diagnosis data were obtained from the INDR.

As previously described [11], since 2012, all health medical organizations in Israel are legally required to report all diabetes cases to the INDR. The INDR is automatically updated daily by the HMO based on patient laboratory data.

For this analysis, we included all pregnant individuals without a prior diabetes diagnosis and who delivered at least one live-born child with a GDM diagnosis. Only the first pregnancy was included for individuals with multiple pregnancies during the study period to ensure the longest follow-up time and prevent selection bias by parity. Follow-up began at the LMP and ended on the date of T2DM diagnosis, the date of data extraction (November 13, 2022), or death, whichever occurred first.

### GDM diagnosis and definitions

By convention and according to the Israeli guidelines, all parturients are recommended to undergo fasting plasma glucose level in the first trimester to exclude overt diabetes (> 125 mg/dL), followed by the two-step approach for GDM diagnosis. Pregnant individuals with diabetes risk factors, such as BMI > 30 kg/m^2^, previous GDM, previous macrosomia, or a strong family history of T2DM, are advised for direct oGTT instead of GCT. GDM is diagnosed if two abnormal oGTT values in oGTT according to the Carpenter–Coustan diagnostic thresholds [12] or if GCT is above 200 mg/dL.

For the INDR, diabetes is defined as meeting one or more of the following criteria: (1) glycated hemoglobin greater than or equal to 6.5% (47.5 mmol/L), (2) serum glucose concentrations greater than or equal to 200 mg/dL (11.1 mmol/L) in two tests performed at an interval of at least 1 month, and (3) three or more purchases of glucose-lowering medications (determined as 3 to avoid misclassification of diabetes). The registry has a sensitivity of 95%, and the positive predictive value is 93% [11].

Obesity was defined as BMI ≥ 30 kg/m^2^. Hypertension was defined as hypertension diagnosis prior to pregnancy. Hyperlipidemia was defined as levels of triglycerides at or above 150 mg/dL or cholesterol level at or above 200 mg/dL from 6 months before or at the first trimester of pregnancy, whenever available.

### Statistical analysis

The study cohort was divided into two groups by fetal sex. At first, we performed univariate analysis to compare maternal characteristics, plasma glucose values, and outcomes between groups. Maternal age and BMI were evaluated both as continuous variables and as categorical variables (with a cutoff of 35 and 40 years for age and 30 kg/m^2^ for BMI). Glucose levels, gestational age at delivery, and time to follow-up were treated as continuous variables, while hypertension, GDM, and T2DM were treated as categorical variables. Data were presented as median [interquartile range (IQR)] for continuous variables and *n* (%) for categorical values. All numbers were rounded to the tenth. Categorical variables were compared using *χ*^2^ tests, and the Mann–Whitney test was used to assess differences for continuous variables. All the tests were two-tailed and *p* < 0.05 was considered statistically significant. Next, we computed a multivariant analysis and the Kaplan–Meier hazard curves for the incidence of T2DM by fetal sex and utilized Cox proportional hazard models to estimate the hazard ratios (HRs) and 95% confidence intervals (CIs) for incident and prediction performance of T2DM over follow-up time with adjustment to confounders.

## Results

**Fig. 1:**
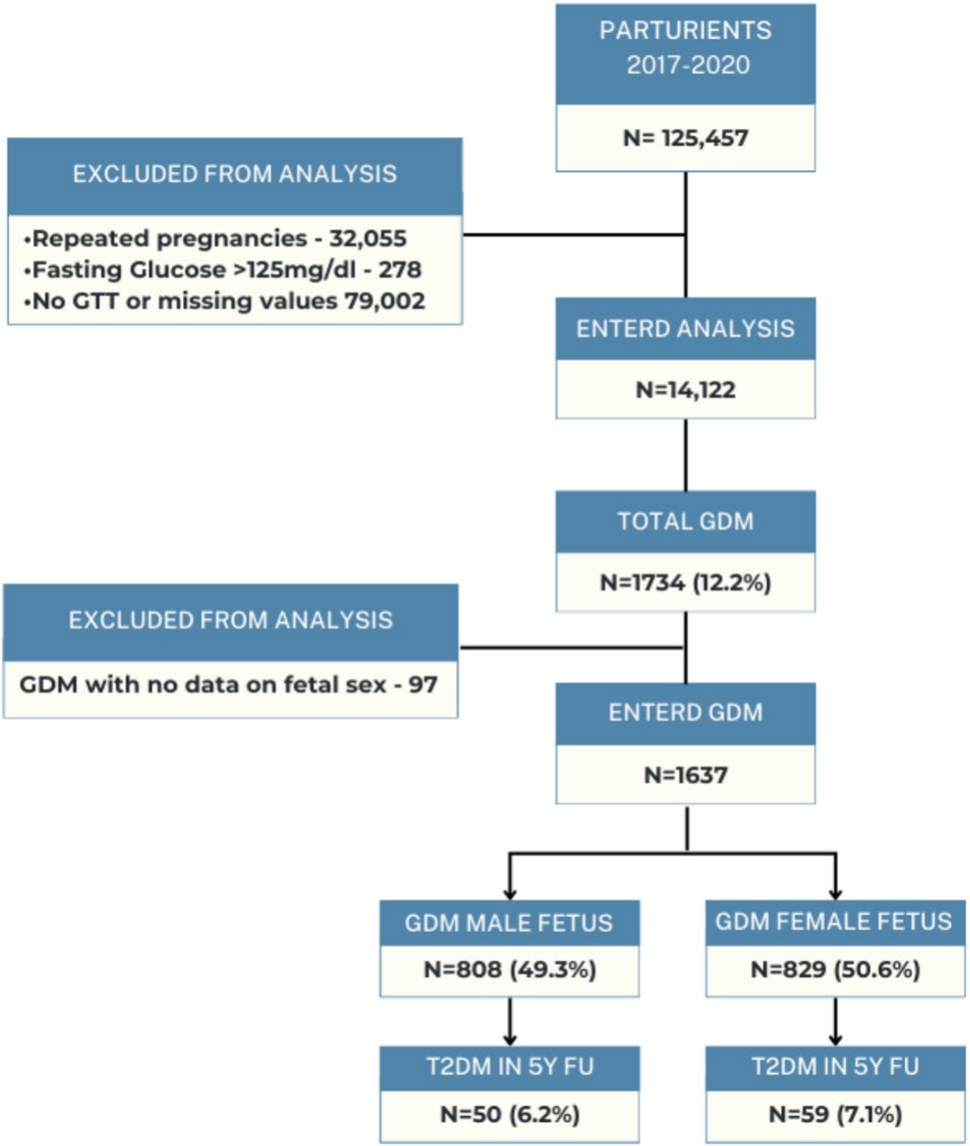
Study cohort

During the study period, 125,457 parturients were recorded into the database. Following exclusion (overt diabetes, no live birth, and no GCT\OGTT data), 14,122 performed oGTT, 1734 (prevalence 12.27%) were diagnosed with GDM, and of them, there were no data on fetal sex of 97 patients. Overall, 829 (47.8%) were female sex pregnancies and 808 (46.5%) were male sex pregnancies (Fig. 1).

Maternal demographic and obstetric characteristics are shown in Table 1. Overall, the median maternal age was 33.2 (28.5–37.7 years), 634 (41.8%) had obesity (BMI ≥ 30 kg/m^2^), and 182 (39.8%) had hyperlipidemia. GDM was diagnosed at median 27.4 (25.9–30.0) gestational weeks and the median follow-up time was 3.9 (2.9–4.9, years). There were no statistically significant differences between pregnant individuals carrying male or female fetuses.

**Table 1:** Baseline demographic and future type 2 diabetes stratified by study groups

	GDM with male fetus *N* = 808	GDM with female fetus *N* = 829	*p* value
Maternal age, years	33.1 (28.5–37.6)	33.3 (28.5–37.5)	0.695
Age ≥ 35 years	325 (40.2%)	352 (42.5%)	0.367
Age ≥ 40 years	104 (12.9%)	110 (13.3%)	0.826
BMI Kg/m2	28.5 (24.6–32.6)	28.1 (24.5–32.7)	0.720
BMI ≥ 30	323 (43.4%)	311 (40.2%)	0.212
Hyperlipidemia^$^	91 (39.4%)	91 (40.3)	0.924
Hypertension	15 (1.9%)	22 (2.7%)	0.320
First-trimester fasting glucose, mg/dL	88 (82–94)	88 (81–95)	0.516
GCT, mg/dL	161 (146–181)	163 (148–179)	0.538
Fasting oGTT, mg/dL	87 (78–96)	87 (78–97)	0.847
1 h oGTT, mg/dL	197 (186–211)	197 (187–214)	0.720
2 h oGTT, mg/dL	170 (158–185)	170 (158–187)	0.642
3 h oGTT, mg/dL	115 (87–140)	112.5 (88–140)	0.990
oGTT week	27.4 (25.9–30)	27.6 (25.9–30.3)	0.308
Gestational age at delivery, weeks	39.1 (38.1–39.9)	39 (38.1–39.9)	0.967
Follow-up time	3.9 (2.9–4.9)	3.9 (2.9–4.9)	0.775
Type 2 DM, 1 st year	1 (0.1%)	4 (0.5%)	0.212
Type 2 DM, 2nd year	16 (1.9%)	12 (1.5%)	0.569
Type 2 DM, 3rd year	31 (3.7%	21 (2.6%)	0.206
Type 2 DM, 4th year	49 (5.9%)	35 (4.3%	0.179
Type 2 DM, cumulative	59 (7.1%)	50 (6.2%)	0.488

Values are presented as median (IQR) for continuous variables and n (%) for categorical values

*BMI* body mass index, *GCT* glucose challenge test, oGTT oral glucose tolerance test, *DM* diabetes mellitus

^$^Available for 457 women

In the univariate analysis during the 5-year follow-up, 109 (6.7%) women developed T2DM. Of them, 59 (7.1%) were carrying male sex pregnancies and 50 (6.2%) were carrying female sex pregnancies (*p* > 0.05).

**Fig. 2:**
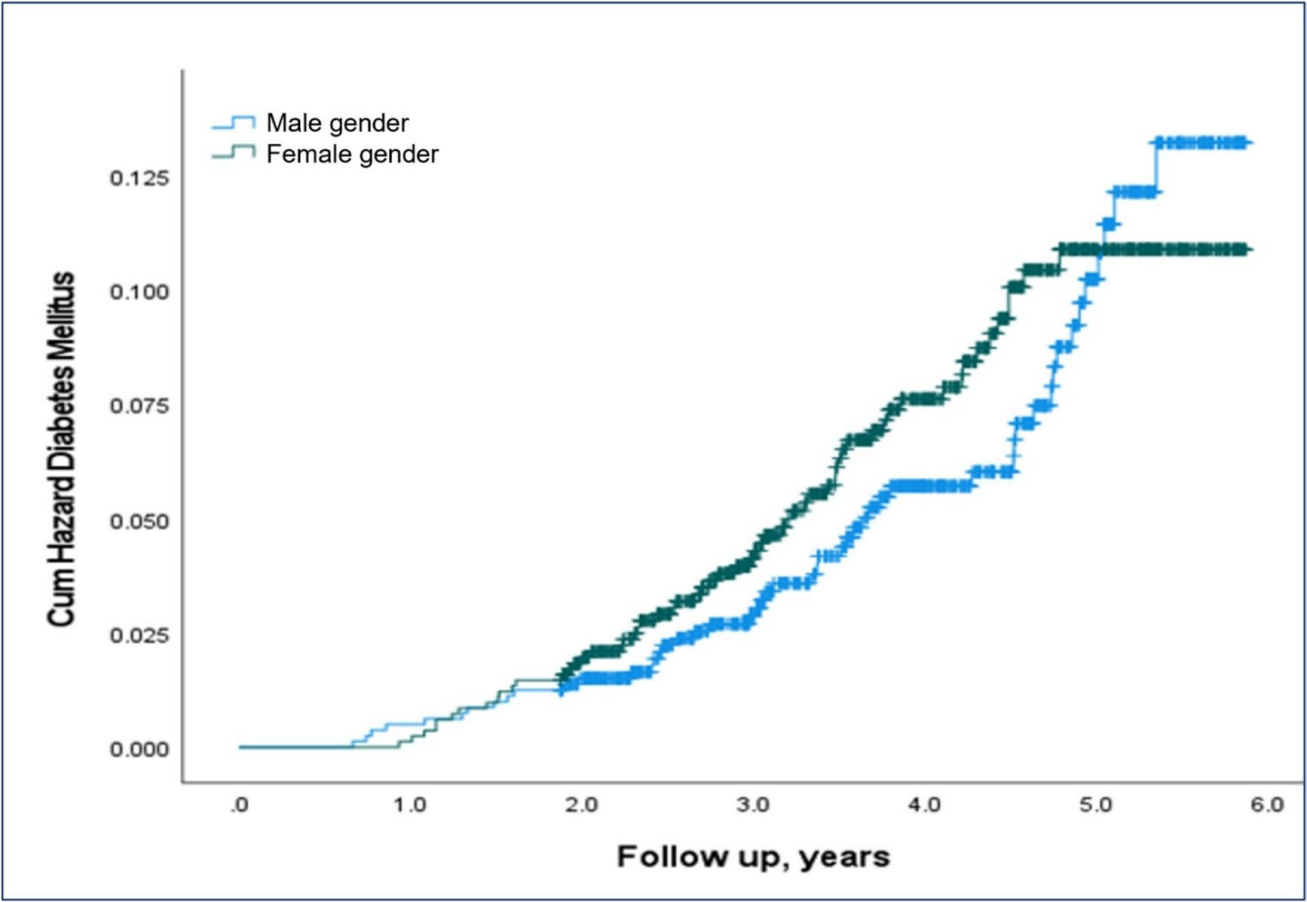
Fetal sex and cumulative risk for type 2 diabetes mellitus**.** Kaplan–Meier hazard curve demonstrating the cumulative risk of type 2 diabetes stratified by fetal sex

Next, we applied survival analysis to account for the development of T2DM over time. Adjusted to maternal age, BMI, and hyperlipidemia, fetal sex had no significant effect on the development of future maternal T2DM (Fig. 2, Table 2).

**Table 2:** Cumulative incidence of type 2 diabetes mellitus during the study period

	aHR	95% CI	*p* value
Maternal age, years	1.073	1.004–1.147	**0.038**
BMI ≥ 30	2.567	1.150–5.730	**0.021**
Hyperlipidemia	0.984	0.463–2.091	0.966
Fetal sex	2.116	0.955–4.687	0.065

*aHR* adjusted hazard ratio, *CI* confidence interval

## Discussion

In our study, we aimed to assess the relationship between fetal sex and the risk of developing T2DM among pregnant individuals with GDM, up to 5 years postpartum.

Our analysis shows that the incidence of T2DM up to 5 years postpartum was similar in pregnant individuals carrying male and female fetuses, with rates of 6.2% and 7.1%, respectively (*p* = 0.488). This lack of statistically significant difference suggests that contrary to previous studies, fetal sex does not have a role in determining the risk of T2DM following GDM.

Previous studies indicate that fetal glucose metabolism is influenced by fetal sex, with women carrying male fetuses demonstrating reduced pancreatic beta-cell function during pregnancy, elevated postprandial glucose levels, and an increased risk of GDM, independent of traditional diabetes risk factors [13]. Additionally, research has identified male fetal sex as an independent risk factor for increased perinatal mortality and morbidity [7]. A meta-analysis revealed that women carrying male fetuses have a 4% higher risk of developing GDM compared to those carrying female fetuses [9]. Giannubilo et al. also demonstrated elevated glucose levels during the oGTT in pregnant individuals carrying male fetuses, greater fetal abdominal fat accumulation, and an increased need for insulin therapy to manage GDM [14].

The pathophysiologic basis of the relationship between fetal sex and diabetes may be related to the degree of insulin resistance and the impairment of maternal β-cell function [14]. Moreover, placentas can also be structurally or functionally different, depending on the sex of the fetus, with different gene, steroid, and protein expressions [14]. Normal β-cell adaptation involves placentally derived maternal hormones, such as human placental lactogens and prolactin, leading to the expansion of β-cell mass and enhanced insulin secretion [15]. A male fetus might induce additional insulin resistance through higher testosterone production in the maternal circulation. However, although the relationship between fetal sex and intrauterine growth has been previously investigated in several studies, the results are somewhat conflicting, probably due to differences in ethnic and obstetric characteristics, study designs, and sample size.

Based on previous studies’ findings, regarding the association between male fetuses and poorer β cell dysfunction [8], it was assumed that male fetuses increased the risk of T2DM or GDM in subsequent pregnancy. However, a large retrospective Canadian study [16] found that in women with GDM, delivery of a girl is associated with a higher risk of early progression to T2DM, compared with having a boy. However, carrying a male fetus is associated with an increased risk of GDM overall but does not increase the likelihood of its recurrence in a second pregnancy. Notably, the median follow-up time was 3.8 years and the rate of GDM was 3.6% in the entire cohort.

These inconsistent findings suggest that the relationship between fetal sex and the development of diabetes during and after pregnancy remains unclear and may not be causally established, as our study implies.

Our study benefits from the large diverse cohort and meticulous data collection. We analyzed a retrospective large database from Meuhedet HMO and cross-referenced it with the INDR. Using data from these two valid nationwide registries, we earned a large, high-quality sample size. This was one of the main strengths of our work and provided us with solid ground for evaluating the long-term impacts of GDM.

However, certain limitations must be acknowledged. The retrospective nature of the study, as well as the relatively short follow-up period of 5 years, may not fully capture the long-term trajectory of T2DM risk in this population. Moreover, we could not account for other maternal risk factors for the development of T2DM such as family history of diabetes. Future studies should consider extending the follow-up duration to better understand the cumulative effects of GDM on metabolic health over a lifetime and provide a more comprehensive understanding of T2DM risk stratified by fetal gender.

In conclusion, while our study did not find significant differences in T2DM risk based on fetal sex among women with GDM, it highlights the necessity for more studies to clarify the complex interplay of factors influencing maternal health outcomes. As the prevalence of GDM continues to rise globally, understanding these dynamics is important for developing targeted preventive strategies and improving health interventions for women.

## Data Availability

Data are available upon request.
